# Combined surgery with 3-in-1 osteosynthesis in congenital pseudarthrosis of the tibia with intact fibula

**DOI:** 10.1186/s13023-020-1330-z

**Published:** 2020-03-02

**Authors:** Yaoxi Liu, Ge Yang, Kun Liu, Jiangyan Wu, Guanghui Zhu, Jin Tang, Yu Zheng, Haibo Mei

**Affiliations:** Department of pediatric orthopaedics, Hunan Children’s Hospital, The Pediatric Academy of University of South China, 86 ziyuan road, yuhua district, Changsha City, Hunan Province 410007 People’s Republic of China

**Keywords:** 3-in-1 osteosynthesis, Congenital pseudarthrosis of tibia, Fibula intact

## Abstract

**Background:**

Re-fracture is the most serious complication in congenital pseudarthrosis of the tibia (CPT). There are reports that children with small cross-sectional areas in the sections of the pseudarthrosis are more prone to re-fracture. Presently, preventing complications is a challenge. Increasing the cross-sectional area in healed segments may reduce the incidence of re-fracture.

**Purpose:**

To elucidate the indications, surgical technique, and outcomes of combined surgery and 3-in-1 osteosynthesis in CPT with intact fibula.

**Methods:**

We retrospectively assessed 17 patients with Crawford Type IV CPT with intact fibula (Type A) who were treated with combined surgical technique and 3-in-1 osteosynthesis between March 2014 and August 2015. The average age of the patients at the time of surgery was 3 years. Incidence of re-fracture, ankle valgus, proximal tibial valgus, and limb length discrepancy (LLD) were investigated over an average follow-up time of 47 months.

**Results:**

Primary union was achieved in all patients. The average time for primary union was 4.9 months. Fifteen (88%) cases showed LLD with an average limb length of 1.6 cm; 6 (35%) cases exhibited tibial valgus with an average tibial valgus deformity of 7.8°; 2 cases had ankle valgus, wherein the ankle valgus deformity was 12° in one and 17° in another; and the cross-sectional area of the bone graft was enlarged to 1.74 times that of the tibia shaft. No case had re-fracture during the follow-up period. Movement of the ankle joint was restored in 16 patients with an average dorsiflexion of 22° and an average plantar flexion of 41°; the function of the ankle joint was normal. One patient had plantar flexion of 20° but did not have dorsiflexion.

**Conclusion:**

Combined surgical technique with 3-in-1 osteosynthesis, which is primarily considered for bone union with a large cross-sectional area, results in a high primary union rate. This can provide satisfactory results in short-term follow-up when treating CPT with intact fibula (Type A).

## Introduction

Presently, the global primary union rate of congenital pseudarthrosis of the tibia (CPT) is relatively high [1–6]. However, preventing complications is challenging. Re-fracture is the most serious complication, with an incidence of 11–68% [7]. Choi et al. [7] reported that children with small cross-sectional areas in the section of the tibial pseudarthrosis are more prone to re-fracture.

Therefore, we designed a modified surgical approach for treating children with CPT with intact fibula (Type A; normal fibular integrity in the presence of established atrophic type CPT [8]) to increase the cross sectional area in the healed segment, thereby reducing the incidence of re-fracture. Presently, increasing the cross-sectional area in the healing segment involves 4-in-1 osteosynthesis along with a shotgun operation [2, 8].

We designed a modified surgical method consisting of an operation [9, 10] combined with 3-in-1 osteosynthesis for the treatment of CPT with intact fibula (Type A). The purpose of this investigation was to introduce the detailed operational procedure of this approach for the treatment of children with CPT with intact fibula and reduce the incidence of re-fracture, ankle valgus, tibial valgus, and tibia length discrepancy.

## Patients and methods

The study was approved by the ethics committee of our institution. Between March 2014 and August 2015, and we assessed 17 patients (12 boys; 5 girls) with Crawford Type IV CPT with intact fibula (Type A) who were treated with combined surgical technique and 3-in-1 osteosynthesis. The surgery was performed on the right leg in 10 patients and on the left leg in 7 patients. Among the 17 patients, 4 had proximal tibia dysplasia [11, 12]. Fifteen patients had no history of previous surgery, and 2 patients had undergone an unsuccessful procedure in another hospital. The average age of patients at the time of surgery was 3 years (1.1–7.7 years). Ten cases exhibited neurofibromatosis (Type 1).

Patients enrolled in this study fulfilled the following criteria: (1) more than 3 years of follow-up data available; (2) pseudarthrosis was located between the middle and distal third of the tibia; (3) operations were performed or supervised by a single surgeon; (4) a complete dataset existed for each patient.

### Surgical technique

#### Harvesting autogenic iliac bone

**Fig. 1:**
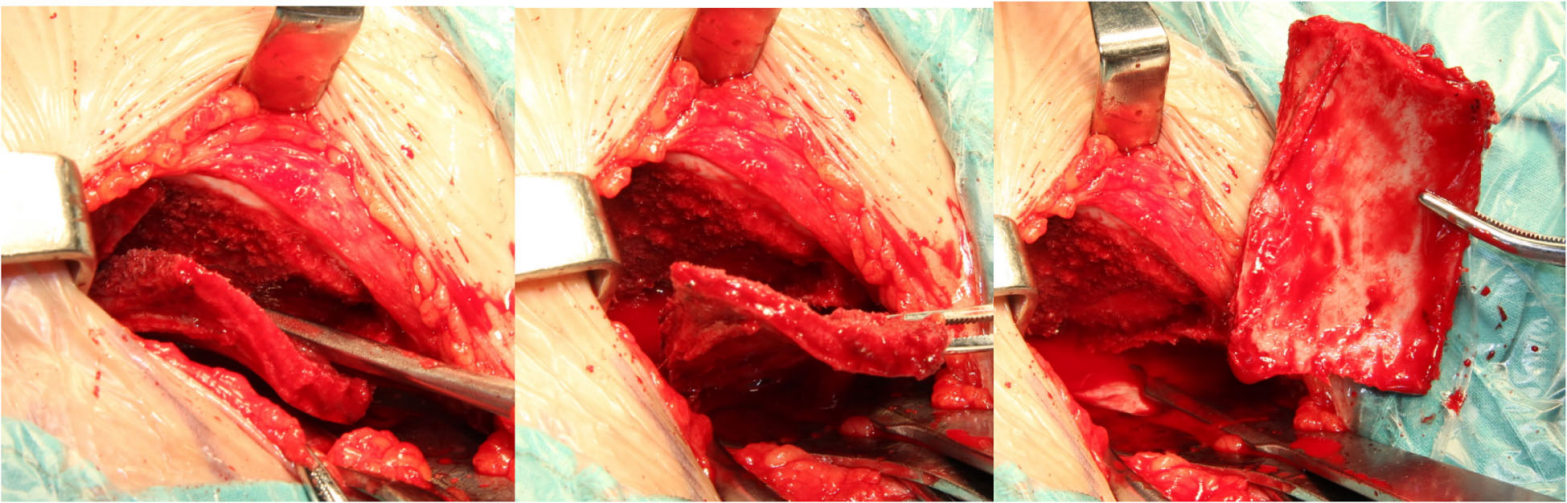
Exposure of outer table of the ilium, harvesting a square of cortex measuring 4 × 3 cm

**Fig. 2:**
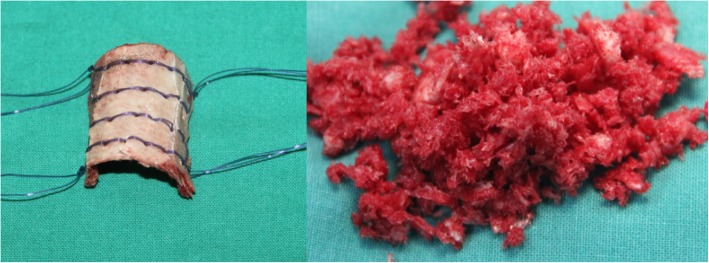
Holes made in the rectangular cortex using Kirschner wire and absorbable sutures. Cancellous bone curetted from supra-acetabular region

Patients were placed in a supine position on a radiolucent operating table with a support beneath the hip. An iliac bone graft was harvested through a straight incision centered over the anterior superior iliac spine. The apophysis of the ilium was split and the outer table of the anterolateral surface of the ilium was exposed sub-periosteally. A rectangular cortex (approximately 4.0 × 3.0 cm; exact size depended on patient’s age) was obtained from the outer table of the ilium, and most of the cancellous bone that was obtained was curetted from the supra-acetabular region, while keeping the inner wall intact. Subsequently, a series of holes were made in the rectangular cortex using a fine Kirschner wire followed by weaving with absorbable sutures to obtain a cylindrical shape for wrapping the graft (Figs. 1 and 2) [9].

#### Proximal osteotomy of the fibula

The longitudinal incision was generally about 3.0 cm at the proximal lateral fibular head. The proximal fibula was osteotomized below 1.5–2.0 cm of the fibular head. The fibula performs subperiosteal osteotomy. The fibula was osteotomized to prevent the high tension resulting from the fusion of the tibia and fibula, and to prevent curving of the fibula when the tibia and fibula fuse. Further, osteotomization can lead to better fusion.

#### Excision of pseudarthrosis, intramedullary rod insertion, and installation of Ilizarov’s fixator

The tibia was approached through an anterior outward straight incision over the site of the pseudarthrosis. The fascia was completely cut to prevent osteofascial compartment. The excised pseudarthrosis exposed the abnormal periosteal surrounding pathologic soft tissues and the partly sclerotic bone ending. At the same time, approximately 6.0 cm of the fibula was freed to facilitate osteosynthesis. The medullary canal of both the proximal and distal tibia fragments were opened with a drill so that a rod could be inserted. The rod was then driven retrogradely into the proximal tibia fragment, which was anatomically aligned to both the coronal and the sagittal planes using intraoperative imaging. After the insertion of the intramedullary rod, an Ilizarov’s fixator was installed with one ring positioned above the site of pseudarthrosis and one below. The tibia and fibula were bundled with absorbable sutures at the proximal and distal end of the tibial pseudarthrosis. When the distal tibia was short, we added a U-ring of calcaneus to improve stability of the external fixation device.

#### 3-in-1 bone osteosynthesis

**Fig. 3:**
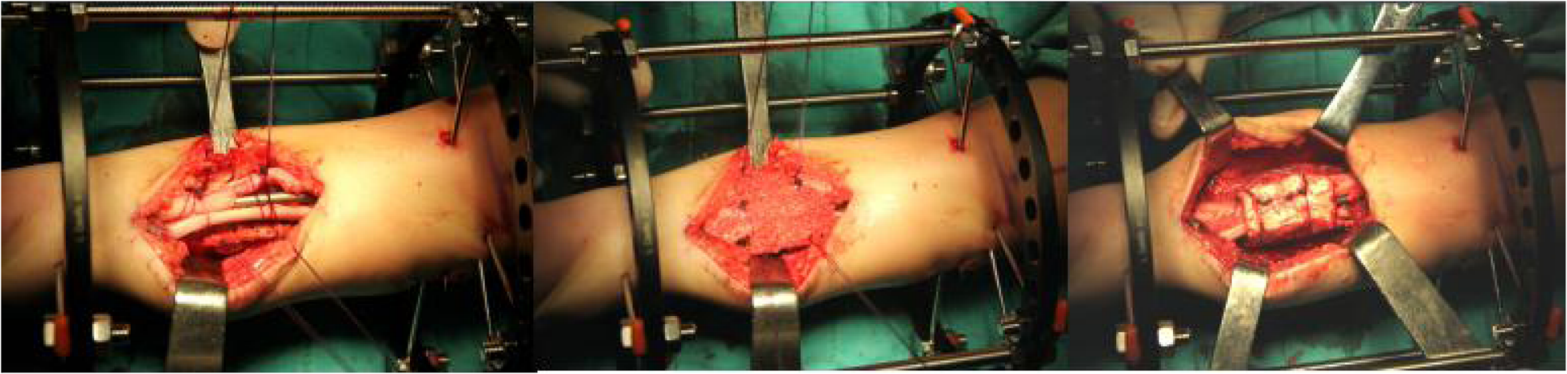
Proximal osteotomy of the fibula, cylindrical cortex wrapped around the proximal and distal segments of the tibia and fibula, cancellous bone compacted circumferentially between the cortex and the pseudarthrosis site of the tibia, wrapping the bone graft, and securing by tying the sutures

3-in-1 osteosynthesis was performed by placing the proximal and distal segments of the tibia and fibula into one bone healing mass. Cylindrical cortex was wrapped around the tibia of the pseudarthrosis site and fibula, cancellous bone was compacted circumferentially between the pseudarthrosis site in the tibia and fibula, and the bone graft was wrapped and secured with sutures. A collagen sponge was placed on the anterior side to restrict cancellous bone displacement (Fig. 3). External fixation was used to improve stability and reliability of pseudarthrosis fixation, and compression was applied at the pseudarthrosis. Compression took place along 3.0–5.0 mm of the pseudarthrosis site.

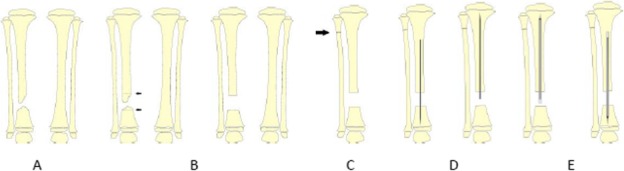



A: CPT of right side B: Excision of pseudarthrosis C: Proximal osteotomy of the fibula D: Kirschner wire as guide wire E: Re-open the medullary canal with a drill.

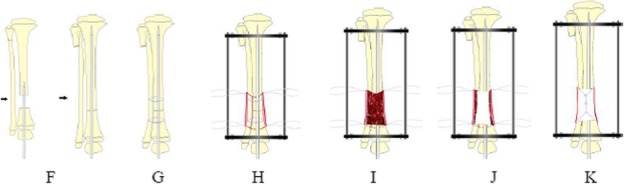



F: Rods inserted G: Binding the tibia and fibula with absorbed suture H: External fixator was applied and the cortex bone was laid blow the tibia and fibula I: Filling the cancellous bone between the tibia and fibula J: A collagen sponge was placed in the anterior side to restrict the cancellous bone displacement K: The wrapping bone graft was secured by tying the sutures.

#### Clinical and X-ray evaluation

Criteria for the healing of CPT were as follows: (1) radiographic union score for tibial score > 8 points [13]; (2) cross-sectional area ratio (calculated as the tibial cross-sectional area of healed area/the average cross-sectional area of the tibia shaft) equal to the diameter of healing at the level of the pseudarthrosis along the AP view × the diameter of healing at the level of the pseudarthrosis along the lateral view [7]; (3) proximal tibia shaft cross-sectional area equal to the proximal tibia shaft diameter along the AP view × the proximal tibia shaft diameter along the lateral view; and (4) the distal tibia shaft cross-sectional area equal to the distal tibia shaft diameter along the AP view × the distal tibia shaft diameter along the lateral view.

#### Post operation management

The needle path was cleaned once every 2 days with normal saline. We observed the clinical manifestation of osteofascial compartment syndrome in all patients. Patients underwent X-ray examination every 2 months. When the pseudarthrosis of the tibia had consolidated, the Ilizarov’s fixator was removed and a long tube-type leg cast was applied for approximately 2 months. After the cast was removed, a protective long leg knee-ankle-foot brace was used to protect the affected extremity during weight-bearing walking. The brace was worn constantly, including during sleep and swimming, until skeletal maturity was attained. The only time the brace was removed was during bathing.

## Results

Seventeen patients with Crawford type IV CPT and intact fibula (Type A) who were treated with combined surgical technique and 3-in-1 osteosynthesis were analyzed. The average age at the time of surgery was 3 years. Among the 17 patients, 4 had proximal tibia dysplasia. Kirschner pin percutaneous osteotomy was used to correct the proximal tibial angle deformity at the vertex of the deformity in 4 patients with proximal tibial dysplasia, so that the intramedullary rods could be successfully inserted into the proximal tibia metaphysis. Tibial length discrepancy was more than 3.0 cm in 2 cases. Tibial lengthening was performed simultaneously. The average surgical time was 4.1 h (3.3–4.2 h), average blood loss was 128 ml (50–400 ml), and 8 cases were infused with 0.75 units of concentric red blood cells during the surgery. No bone substitutes were added to any patients.

None of the patients developed pin tract infection. There were no osteofascial compartment syndromes in any patients. The mean retention time of external fixation was 5.1 months (4.1–7.8 months) and the average time of tube-type cast fixation was 2.3 months (2–4 months). No cases were mature at the time of the last follow-up. In all cases, the distal position of the distal intramedullary rod was adjusted to the distal epiphysis of the tibia.

**Fig. 4:**
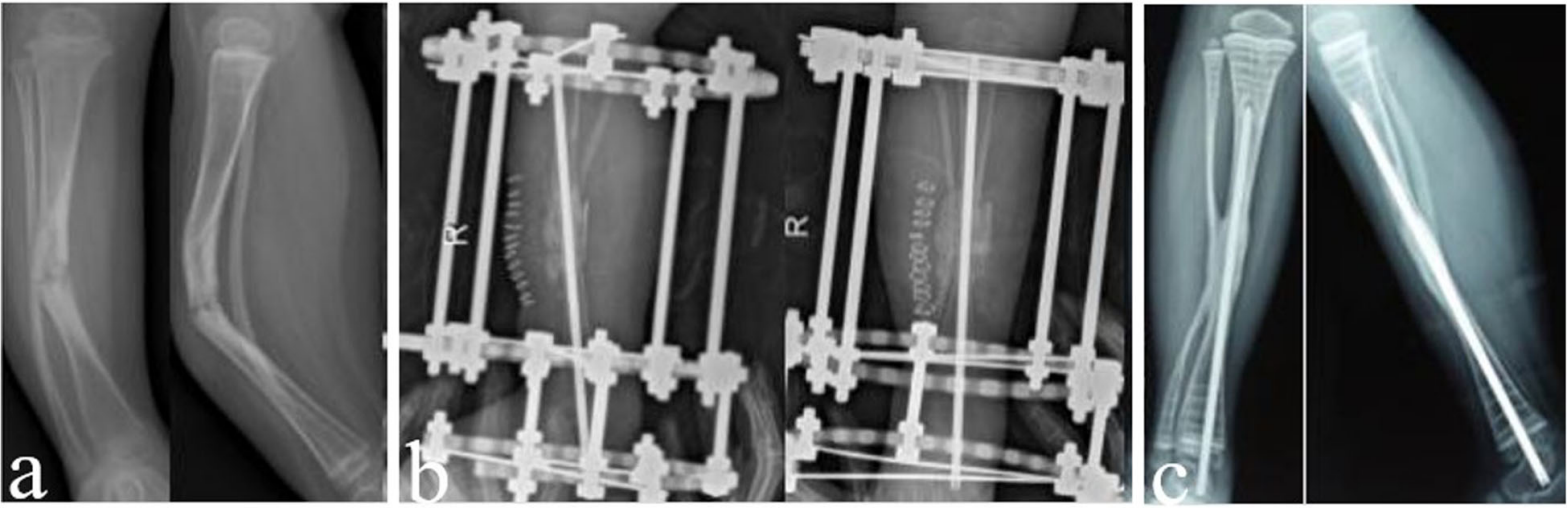
**a** Preoperative anteroposterior and lateral radiographs. **b** X-ray radiograph one week post-operation. **c** Twenty-three months after the operation, X-ray shows that the cross sectional area of the healed segment was enlarged

Primary healing was achieved in all 17 cases, with a mean follow-up time of 47 months (36–53 months) (Fig. 4). The average healing time was 4.9 months (4.1–7.8 months), and the primary healing rate was 100%. The cross-sectional area of the tibial pseudarthrosis healing segment increased 1.74 times (1.14–2.60 times) compared to that of the tibial shaft.

All 17 patients were able to walk independently. Sixteen patients had restored movement of ankle joint, with an average dorsiflexion of 22^o^ (20–30^o^) an average plantar flexion of 41^o^ (40–50^o^), and normal function of the ankle joint. One case had plantar flexion of 20^o^ but no dorsiflexion. Fifteen (88%) cases showed LLD with an average limb length of 1.6 cm (0.4–2.9 cm).

**Fig. 5:**
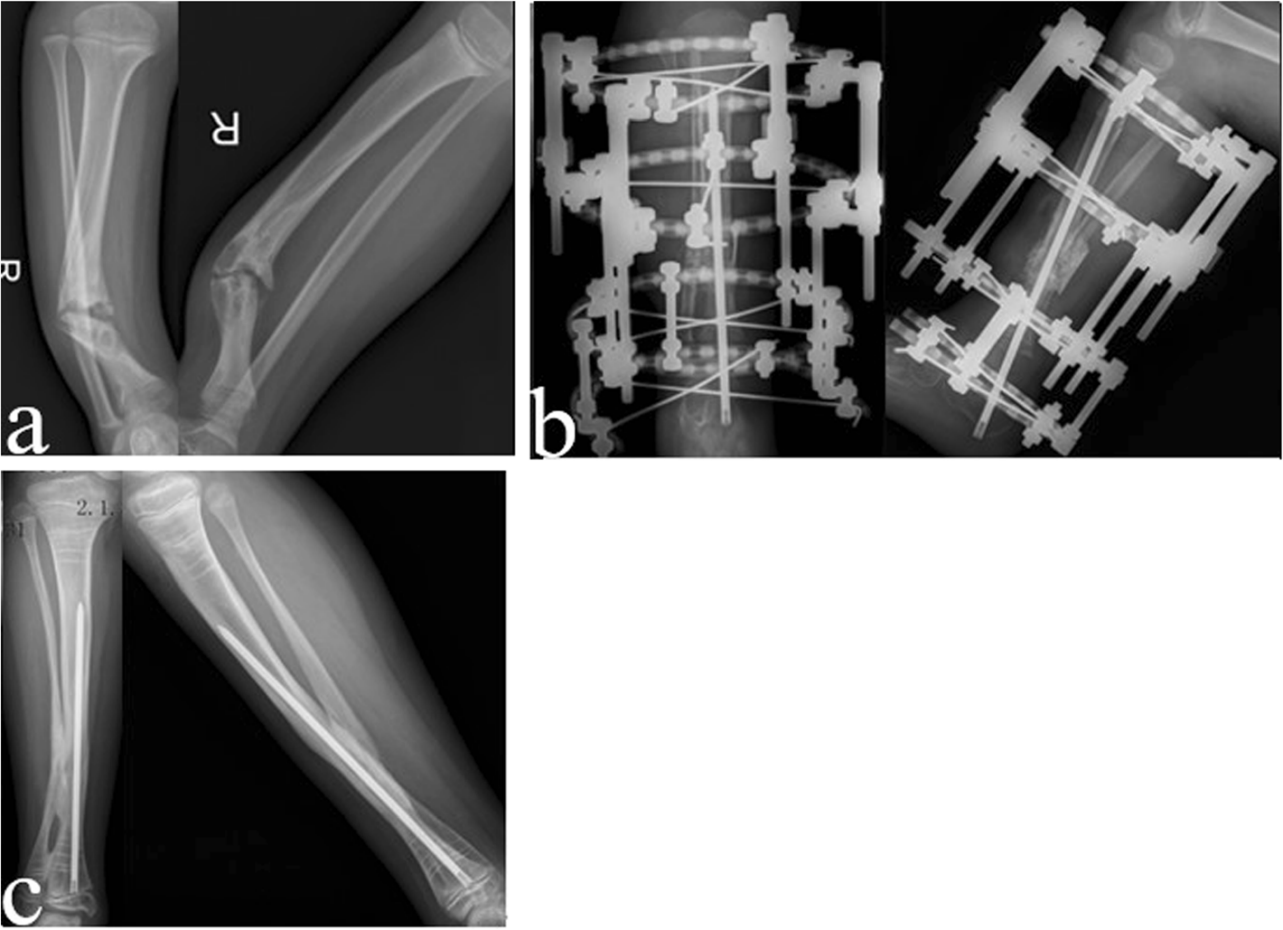
**a** Preoperative anteroposterior and lateral radiographs. **b** The X-ray radiograph, 4 days of post-operation. **c** Forty-eight months after the operation, X-ray shows that the tibia and fibula were well fused

Six cases (35%) had tibial valgus. Half epiphysis plate block was performed on the medial tibial “8” plate, and the proximal tibia valgus was corrected. No re-fracture occurred in any patient. In 2 cases, ankle valgus occurred, one case with 12° ankle valgus, and the other case with 17° ankle valgus. Two patients with ankle valgus were corrected by half epiphysis block (Figs. 4 and 5) (Table 1).

**Table 1:** The situation of CPT patients

Number	follow-up (months)	age	surgery history	NF1	union time	R (CSA)	PTV	AV	LLD	Refracture
N1	52 M	2.6Y	N	N	4.4 M	1.43	N	N	1.6 cm	N
N2	52 M	4.8 Y	N	N	4.2 M	1.87	5°	12°	0 cm	N
N3	51 M	2.7 Y	N	N	6.6 M	1.63	N	N	1.5 cm	N
N4	51 M	1.1 Y	N	N	4.1 M	1.57	7°	N	1.2 cm	N
N5	52 M	1.4 Y	N	Y	4.0 M	1.70	N	N	0.6 cm	N
N6	53 M	7.7 Y	Y	N	5.3 M	1.32	N	N	1.7 cm	N
N7	41 M	2.2 Y	N	N	4.2 M	2.60	N	N	1.5 cm	N
N8	36 M	3.3 Y	N	Y	4.1 M	1.80	N	N	2.6 cm	N
N9	41 M	3.9 Y	N	Y	4.8 M	1.48	N	N	0.4 cm	N
N10	39 M	3.2 Y	N	Y	4.3 M	2.16	N	N	1.2 cm	N
N11	45 M	2.3 Y	N	Y	4.2 M	1.92	N	N	2.0 cm	N
N12	46 M	4.0 Y	N	Y	5.7 M	1.38	5°	N	0.8 cm	N
N13	47 M	2.3 Y	N	N	4.1 M	1.93	N	N	2.4 cm	N
N14	46 M	3.7 Y	N	Y	4.4 M	2.49	9°	N	2.2 cm	N
N15	48 M	1.3 Y	N	Y	7.8 M	1.33	N	N	0 cm	N
N16	51 M	1.9 Y	Y	Y	4.6 M	1.77	16°	N	2.9 cm	N
N17	50 M	1.7 Y	N	Y	4.6 M	1.14	5°	17°	1.3 cm	N

Note: *NF1* neurofibromatosis, *AV* ankle valgus, *PTV* proximal tibia valgus, *LLD* lower limb discrepancy, *CSA* cross sectional area, *R* ratio. *Y* Yes, *N* No

## Discussion

Re-fracture is the most serious complication in CPT patients following the achievement of primary union, and a small cross-sectional area of the pseudarthrosis is a risk factor for re-fracture. Maximizing the tibial cross-sectional area of the healed segment could reduce the rate of re-fracture in CPT. Maximizing the healing cross sectional area is considered one of the most important principles in the treatment of CPT [14].

Re-fracture is the most serious complication, with an incidence of 11–68% [7]. In 2011, Choi et al. [8] reported the effect of 4-in-1 osteosynthesis and other surgical methods (distal tibia and fibula fusion) in 13 cases of atrophic CPT with B2 fibular pseudarthrosis. In that study, 8 patients received 4-in-1 bone osteosynthesis and 5 underwent other surgical methods (control group). Post-operative follow-up showed that the 8 patients who had 4-in-1 bone osteosynthesis did not experience re-fracture. In the control group, only one patient did not experience re-fracture. After 7.4 years, re-fracture rate in the 4-in-1 osteosynthesis group was 0%, while the control group had a progressive increase: 40% at 1.8 years, and 80% at 2.7 years.

In 2012, Paley [2] reported periosteal transplantation, autologous cancellous bone grafting, intramedullary rod, external fixation, tibia and fibula fusion, BP, and BMP in 15 children with CPT, of which all achieved union. The average follow-up period was 2 years without re-fracture.

Here, in the case of the fibula integrity patients, 3-in-1 bone osteosynthesis was applied along with combined surgery to increase the cross-sectional area of the healed segment. Seventeen patients had no re-fracture after treatment, similar to the results of the studies described above. One patient experienced plantar flexion of 20° but no dorsiflexion. This may be related to the intramedullary rod fixing the ankle joint in the plantar flexion location. Ankle valgus occurred in 2 cases, which may be related to the intramedullary rod not being placed through the center of ankle joint. In addition, 6 patients had tibia valgus, the reasons for which are unclear and warrant further investigation.

### Differences and similarities between 3-in-1 and 4-in-1 bone osteosynthesis

**Fig. 6:**
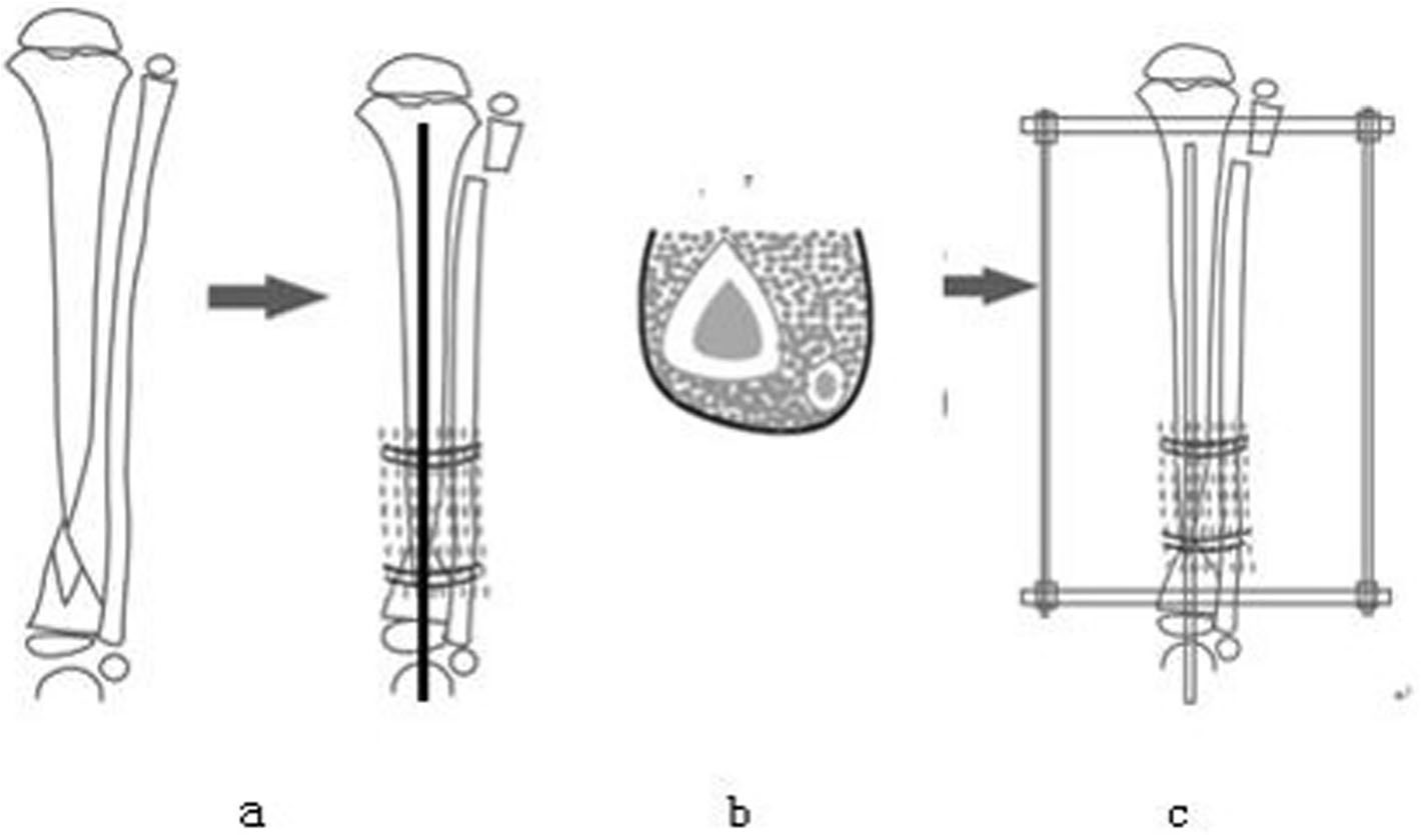
**a** Suturing the tibial pseudarthrosis at the ends of the tibia and fibula. **b** Filling the cancellous bone between the tibia and fibula. **c** Wrapping the bone graft and securing by tying the sutures

**Fig. 7:**
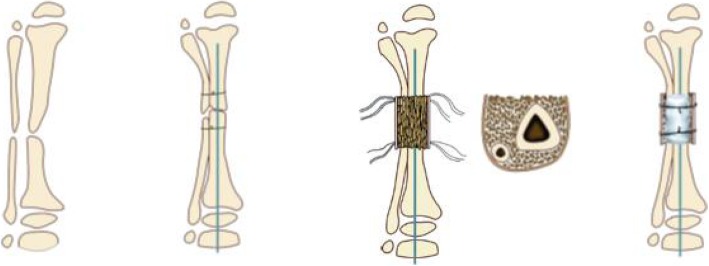
A schematic diagram of 4-in-1 bone osteosynthesis

Generous autogenous bone grafting is an essential part of 3-in-1 and 4-in-1 bone osteosynthesis (Figs. 6 and 7). Copious cancellous chips are places in the intervening gap between the tibia and fibula. In 4-in-1 bone osteosynthesis, large autologous iliac plates are used to wrap the pseudarthrosis of the tibia and fibula. This procedure allows all 4 segments of the tibia and fibula to be placed in a single fusion mass. This procedure is suitable for children with tibia and fibula pseudarthrosis at the same level.

However, 3-in-1 bone osteosynthesis is possible when the proximal fibula is osteotomized. The tibial pseudarthrosis and fibula undergo osteosynthesis with a rectangular cortical bone and cancellous bone. The anterior side is covered with a collagen sponge to reduce the displacement of the grafts. This procedure allows all 3 segments of the tibia and fibula to be placed in a single fusion mass. This procedure is suitable for children with fibula integrity (Type A). Both methods aim to increase the cross-sectional area of the healed segment to prevent re-fractures.

### Innovative aspects of the technique

3-in-1 bone osteosynthesis has unique advantages: it can maximize the cross-sectional area of the healed segment, as the proximal osteotomy of the fibula is conducive to osteosynthesis of the fibula and tibia. It can also prevent the fibula from bending when the Ilizarov external fixator is pressurized between the distal and proximal tibia pseudarthrosis. Larger cross-sectional areas of healed segment can better resist mechanical stress and prevent re-fracture. Further, patients are less likely to experience ankle valgus.

### Precautions surrounding application of 3-in-1 bone osteosynthesis

There are several precautions surrounding the use of this approach. (1) The proximal osteotomy of the fibula has strong healing ability, and does not affect the stability of the lateral malleolus. (2) Exposing the anterior tibial vascular nerve bundle, cutting the interosseous membrane and the peroneal periosteum, freeing fibula at least 6 cm, binding the tibia and fibula tightly, and fixing it with No. 1 absorbable sutures. (3) When the intramedullary rod is pushed into the distal and proximal tibia, it must be kept at the central axis of tibia to reduce the incidence of proximal tibia and ankle valgus. If the tibia is curved, this increases the difficulty of maintaining the anatomic axis when the intramedullary rod is pushed into the shaft. In this situation, closed osteotomy of the proximal tibia (which can be percutaneous drilled by Kirshner wire) can be used to restore the axis of the tibia. In this study, proximal tibia valgus occurred in 6 cases, with an incidence of 35% (6/17), and ankle valgus occurred in two cases. Ankle valgus may be related to the intramedullary rod not going through the center of the ankle joint. All cases were corrected by postoperative epiphyseal block.

### Potential problems of 3-in-1 bone osteosynthesis

Some potential adverse effects of this approach are difficult to avoid. It is important to achieve union and achieve function without activity restrictions by the time of skeletal maturity, with the use of a few surgical procedures as possible. The potential impact of 3-in-1 bone osteosynthesis on the growth of the tibia and fibula remains unclear. This could affect the formation of load-bearing bones. The altered growth patterns of the tibia and fibula after tibiofibular synostosis in children can result in the prominence of the fibular head and relative shortening of the lateral malleolus. Although adverse effects of these changes have not yet been observed, it is necessary to perform long-term follow-up and observation to determine whether osteoarthritis, the ratio of the length of tibia and fibula, and movement of the knee and ankle joint are affected in the longer term.

The present study has a few limitations. These include the small sample size and retrospective study design. Further, follow-up periods were not sufficiently long in order to determine the long-term re-fracture and ankle valgus rates.

## Conclusion

The results of early follow-ups showed that 3-in-1 bone osteosynthesis can increase the cross-sectional area of healing tibial pseudarthrosis and reduce the incidence of re-fracture. The rate of primary healing was high. Thus, 3-in-1 bone osteosynthesis could be an effective approach for the treatment of CPT with intact fibula (Type A). This represents a modified surgical treatment option for this condition.

## Data Availability

All data generated or analyzed during this study are included in this published article and its additional files.

## Abbreviations

CPT: Congenital pseudarthrosis of the tibia
NF1: Neurofibromatosis type 1
